# Radiomics-driven and explainable machine learning for rapid characterization of Fusarium wilt and Black Sigatoka in banana crops

**DOI:** 10.3389/frai.2026.1861135

**Published:** 2026-07-28

**Authors:** Rodne Andrés Quijije, Enrique Pelaez, Ana Tapia-Rosero, Francis R. Loayza, Edwin Valarezo, Gabriel Herrera-Perez, Juan Pisco-Jordán, Lus Ramos-Pozo, Isaac Leon, Freddy Magdama, Juan Manuel Cevallos-Cevallos

**Affiliations:** Facultad de Ingeniería en Electricidad y Computación (FIEC), Escuela Superior Politécnica del Litoral, ESPOL, Campus Gustavo Galindo, Guayaquil, Ecuador

**Keywords:** black sigatoka, explainable AI, Fusarium wilt, machine learning, precision agriculture, probability calibration, radiomics, random forest

## Abstract

**Introduction:**

Banana production is increasingly threatened by fungal diseases such as Fusarium wilt and Black Sigatoka, posing severe risks to food security and agricultural economies. Recent image-based approaches using deep learning have shown high predictive capacity for plant disease recognition; however, their limited transparency, calibration uncertainty, and sensitivity to domain shifts can restrict their use in decision-support workflows that require auditability.

**Methods:**

This study proposes an interpretable and calibrated Artificial Intelligence framework for multiclass banana disease-pattern characterization based on radiomic feature analysis of RGB leaf images. Radiomic features were extracted from HSV-segmented banana leaf regions, resulting in a dataset of 14,763 samples characterized by 103 quantitative descriptors and labeled as Healthy, Sigatoka, or Fusarium wilt race 1.

**Results:**

Among the evaluated radiomics classifiers, the calibrated Random Forest achieved accuracy = 0.85, balanced accuracy = 0.84, macro-F1 = 0.84, and macro ROC-AUC OvR = 0.95 on the held-out test set. Bootstrap analysis yielded 95% confidence intervals of [0.8406, 0.8691] for accuracy and [0.8306, 0.8606] for balanced accuracy. Three deep learning baselines trained on the same partition achieved higher predictive performance: MobileNetV3 with accuracy = 0.96, macro-F1 = 0.95, and macro ROC-AUC OvR = 0.97; EfficientNet with accuracy = 0.96, macro-F1 = 0.97, and macro ROC-AUC OvR = 0.96; and ResNet-18 with accuracy = 0.96, macro-F1 = 0.96, and macro ROC-AUC OvR = 0.96.

**Discussion:**

The CNNs produced strong classification performance on the evaluated repositories, and the radiomics approach demonstrated to be a complementary interpretable and explainable calibrated reference model. SHAP, LIME, permutation importance, accumulated local effects, calibration curves, and Brier score decomposition supported feature-level inspection of the final model.

## 1 Introduction

Banana (*Musa* spp.) is one of the most important staple and export crops worldwide, playing a critical role in food security and socioeconomic stability across tropical and subtropical regions (Miao et al., 2025). Ecuador is one of the world's leading exporters of bananas, and the crop represents a strategic pillar of its national agricultural economy (Food and Agriculture Organization of the United Nations, 2026). However, banana production is increasingly threatened by fungal diseases that compromise yield, fruit quality, and plantation sustainability, most notably Fusarium wilt and Black Sigatoka (Van Westerhoven et al., 2022). The growing pressure exerted by these diseases has intensified the need for rapid, early, accurate, scalable, and interpretable diagnostic tools capable of supporting timely phytosanitary interventions.

Fusarium wilt of banana, caused by *Fusarium oxysporum* f. sp. *cubense*, is considered one of the most destructive plant diseases globally because of its soilborne nature, long persistence, and the lack of effective chemical control (Ritter et al., 2025). Of particular concern is Tropical Race 4 (TR4), which has spread across Asia, Africa, and Latin America in recent years (Munhoz et al., 2024). In Ecuador, recent phytosanitary alerts and institutional containment actions have reinforced the need for surveillance technologies that support risk prioritization, field inspection, and confirmatory laboratory workflows as stated by the Ecuadorian agricultural authority Agrocalidad (2026). Although these technologies are not intended to replace official diagnosis, they can help to enhance disease monitoring, the recognition of disease patterns, and prioritize suspect cases for expert inspection and laboratory testing (FAO, 2023; FreshPlaza, 2025).

Black Sigatoka, caused by *Pseudocercospora fijiensis*, represents another major fungal threat, leading to leaf necrosis, reduced photosynthetic capacity, and yield losses if not properly managed (Ploetz, 2015). Unlike Fusarium wilt, Sigatoka symptoms are primarily foliar and visually detectable; however, symptom progression varies with environmental conditions, disease stage, cultivar, and management practice, making manual recognition subjective and error prone. In addition, visual overlap among disease-related leaf alterations may lead to delayed response or incorrect operational actions (Ploetz, 2015; Barbedo, 2018).

Traditional disease identification in banana plantations relies heavily on expert visual inspection and laboratory-based confirmation. While effective, these approaches are time consuming, costly, and difficult to deploy continuously across large agricultural areas, particularly when diseases such as Foc TR4 require coordinated biosecurity, surveillance, and confirmatory response workflows (Munhoz et al., 2024; Agrocalidad, 2026). Artificial Intelligence (AI) and Machine Learning (ML) have therefore emerged as tools for automated analysis of visual and spectral data in plant disease detection (Shafay et al., 2025; Nyawose et al., 2025). Deep learning (DL) architectures, including convolutional neural networks (CNNs), MobileNets, Vision Transformers, and emerging foundation or vision-language models, can achieve strong performance in plant disease recognition from RGB, UAV, multispectral, or hyperspectral imagery (Linero-Ramos et al., 2024; Shafay et al., 2025; Nyawose et al., 2025). Nevertheless, high predictive accuracy alone is not sufficient for phytosanitary decision support: transparency, probability calibration, leakage-aware validation, interpretability, explainability and external testing are also required (Joeres et al., 2025; Ahmed et al., 2025; Tahir et al., 2025).

Radiomics is an approach for extracting quantitative descriptors that characterize intensity, shape, and texture patterns from images (Gillies et al., 2016). Originally developed in medical imaging, radiomics is conceptually aligned with image-based plant phenotyping because both aim to convert image patterns into quantitative, analyzable traits (Singh et al., 2016). Texture descriptors derived from gray-level co-occurrence matrices (GLCM), gray-level run-length matrices (GLRLM), gray-level size-zone matrices (GLSZM), gray-level dependence matrices (GLDM), and neighboring gray-tone difference matrices (NGTDM) can capture lesion heterogeneity, tissue irregularity, and spatial organization; these feature families are widely used in standardized radiomics implementations such as PyRadiomics and follow IBSI-oriented definitions (PyRadiomics Community, 2024; Aguirre-Meneses et al., 2025). When combined with classical ML classifiers, radiomics enables models whose predictions can be inspected at the feature level using permutation importance, SHAP, LIME, and accumulated local effects (Ahmed et al., 2025; Tahir et al., 2025).

The methodological gap addressed in this study is not the absence of high-accuracy image classifiers. Rather, the gap lies in the limited availability of calibrated, leakage-aware, and feature-level interpretable baselines for multiclass banana disease-pattern characterization involving Healthy, Sigatoka, and Fusarium wilt categories. This study evaluates a radiomics-based ML framework together with three CNN baselines (MobileNetV3, EfficientNet, and ResNet-18), to quantify the accuracy–interpretability trade-off under the same data partition. The radiomics model is positioned as a complementary, interpretable and auditable reference model, whereas the CNN baselines establish strong performance image-based benchmarks.

The remainder of this paper is organized as follows. Section 2 reviews recent AI-based approaches for plant disease characterization, including DL, interpretable models, calibration, and leakage-aware validation. Section 3 describes the data sources, leakage-aware partitioning, HSV-based segmentation, radiomic feature extraction, feature preprocessing, classical classifiers, CNN baselines, probability calibration, and statistical analyses. Section 4 presents the comparative results, bootstrap confidence intervals, McNemar testing, CNN baseline comparison, calibration assessment, and explainability analyses. Section 5 discusses the biological plausibility, operational implications, domain-shift limitations, and deployment constraints. Section 6 concludes with future directions for Ecuadorian field validation and hybrid interpretable DL systems.

## 2 Related work

### 2.1 AI-based approaches for plant disease characterization

Fusarium wilt, in particular TR4, has attracted increasing research attention because of its rapid spread and impact on banana production (Ploetz, 2015). Existing characterization strategies focus primarily on molecular diagnostics, soil assays, and expert visual inspection (Ploetz, 2015). While these methods are reliable, they are not easily scalable for continuous field monitoring. Recent work on Fusarium TR4 in Latin America and the Caribbean has emphasized that effective management requires surveillance, biosecurity, containment, resistant cultivars, and coordinated regional response strategies (Munhoz et al., 2024). In addition, the socioeconomic effects of TR4 on banana producers in Latin America further reinforce the need for scalable monitoring and early-warning tools (Ritter et al., 2025). AI-driven approaches for Fusarium wilt characterization remain more limited than those for foliar diseases, and many image-based studies do not explicitly address differentiation between Fusarium wilt and other diseases with overlapping visual characteristics.

Black Sigatoka has been more extensively studied using image-based techniques, but many studies address binary classification or disease severity estimation rather than multiclass differentiation among healthy leaves and multiple disease types. Recent studies using UAV-based multispectral imagery and DL have demonstrated the feasibility of plantation-level monitoring; however, these approaches remain sensitive to dataset scale, geographic variability, acquisition devices, and environmental conditions (Linero-Ramos et al., 2024). These limitations are relevant for banana disease recognition because cultivar diversity, field management, illumination, canopy structure, and regional disease pressure can affect the visual appearance of symptoms and the generalization of image-based models.

AI techniques have been explored for automating the recognition of disease patterns, mainly based on computer vision and using ML techniques. Early approaches focused on handcrafted features combined with classical classifiers, and recent reviews of plant disease detection emphasize that DL has become a dominant modeling paradigm because of its capacity to learn discriminative visual features directly from images (Shafay et al., 2025; Nyawose et al., 2025). Compact CNNs such as MobileNet variants are particularly relevant for agricultural applications because they reduce parameter count and computational requirements relative to larger architectures, whereas EfficientNet and ResNet families remain common image-classification benchmarks. At the same time, recent literature highlights continuing challenges in cross-domain generalization, benchmark design, data leakage, annotation quality, and interpretability (Shafay et al., 2025; Nyawose et al., 2025).

CNN-based approaches have been applied to detect foliar diseases such as Black Sigatoka, using leaf images captured under natural illumination. Previous studies (Lundberg and Lee, 2017; Ploetz, 2015) have reported high classification accuracy using architectures such as VGG, ResNet, MobileNet, and related CNN variants, showing the potential of DL for large-scale disease monitoring (Shafay et al., 2025; Nyawose et al., 2025). UAV-based and multispectral imaging combined with deep neural networks have further extended these capabilities to plantation-level analysis, including Black Sigatoka monitoring in banana crops (Ribeiro et al., 2016; Linero-Ramos et al., 2024). However, these models often require sufficiently large and well-annotated datasets, and their performance may be sensitive to domain shifts caused by illumination, background variability, acquisition devices, geographic conditions, and canopy structure. In addition, many DL models operate through latent feature representations, which can limit direct feature-level interpretability and complicate adoption in decision-support or regulatory contexts (Ahmed et al., 2025; Tahir et al., 2025).

Parallel to DL developments, classical ML methods using handcrafted or radiomic descriptors remain relevant, particularly in scenarios where calibration, computational constraints, auditability, or explainability are important. Texture-based descriptors, including Haralick features derived from gray-level co-occurrence matrices (GLCM), have been widely used to characterize plant disease symptoms because they capture spatial heterogeneity and lesion structure (Haralick et al., 1973; Materka and Strzelecki, 1998; Gillies et al., 2016). These approaches are generally more transparent at the feature level than end-to-end DL models, although they may provide lower raw predictive accuracy. This motivates a direct comparison between calibrated radiomics models and CNN baselines under the same data partition, allowing the accuracy–interpretability trade-off to be quantified empirically.

### 2.2 Radiomics, calibration, and explainability in agricultural AI

Radiomics extends traditional texture analysis by extracting a large set of quantitative descriptors that characterize intensity distributions, shape, spatial heterogeneity, and multiscale texture (Gillies et al., 2016). Originally developed in medical imaging, radiomics has recently been adopted in agricultural and plant phenotyping contexts, where subtle morphological and textural changes may reflect disease progression, stress responses, or tissue degradation (Shafay et al., 2025; Nyawose et al., 2025). These descriptors provide an intermediate representation between raw image pixels and model predictions, enabling quantitative characterization of visual patterns that may otherwise be difficult to inspect directly.

Recent research has demonstrated the utility of radiomic and texture-based features, such as first-order statistics, shape descriptors, GLCM, GLRLM, GLSZM, GLDM, NGTDM, and wavelet-derived descriptors, for characterizing heterogeneous image patterns associated with plant stress and disease. Foundational texture descriptors (Haralick et al., 1973; Materka and Strzelecki, 1998), including Haralick features derived from gray-level co-occurrence matrices, remain relevant for methodological definitions because they capture spatial heterogeneity and local gray-level relationships. In plant pathology, these feature families are useful because fungal infections can induce lesion coalescence, tissue irregularity, necrotic patterns, and non-uniform spatial organization, all of which can be represented through quantitative shape and texture descriptors.

Beyond predictive accuracy, recent research also emphasizes the importance of model calibration and interpretability for deploying AI systems in high-stakes or decision-support domains, including agriculture (Ahmed et al., 2025; Tahir et al., 2025). Poorly calibrated models may produce overconfident predictions, leading to inappropriate prioritization, inspection, or management decisions. Techniques such as Isotonic Regression (Niculescu-Mizil and Caruana, 2005) and Platt scaling (Niculescu-Mizil and Caruana, 2005) have been proposed to improve probability reliability when predicted probabilities are used for risk-aware decision support (Niculescu-Mizil and Caruana, 2005; Dankowski and Ziegler, 2016). In phytosanitary workflows, calibration is relevant because model outputs may be used to define inspection thresholds, prioritize uncertain cases, or communicate confidence to agronomists and plant-health authorities.

Explainable AI (XAI) methods, including Permutation Importance, SHAP, LIME, and Accumulated Local Effects (ALE), are increasingly used to interpret model behavior and identify features associated with model predictions (Lundberg and Lee, 2017; Ribeiro et al., 2016; Molnar, 2023). Recent agricultural AI literature has further emphasized the role of XAI in improving transparency, interpretability, and trust in AI-driven decision-making systems (Ahmed et al., 2025; Tahir et al., 2025). These techniques are particularly valuable in radiomics-based models because explanations can be linked to explicit image descriptors such as perimeter, sphericity, intensity percentiles, and texture non-uniformity. This feature-level interpretation distinguishes radiomics from end-to-end CNN models, where explanations often require post-hoc saliency or attribution methods over latent representations.

Recent work on data splitting in biological ML has also shown that random or naïve partitions may inflate performance when related biological entities, duplicated observations, or highly similar samples appear in both training and test sets. (Joeres et al. 2025) introduced DataSAIL as a leakage-reduced splitting strategy intended to enable more realistic evaluation of ML models in biological data, particularly under out-of-distribution settings. This issue is relevant for agricultural image datasets because images may share acquisition conditions, plant identity, plot identity, or visually similar backgrounds. Therefore, leakage-aware validation is necessary to avoid overly optimistic performance estimates and to better approximate deployment conditions.

While DL approaches dominate recent studies on plant disease recognition, there remains a need for calibrated, leakage-aware, and feature-level interpretable frameworks tailored to multiclass banana disease-pattern characterization. Radiomics-based ML approaches provide a complementary reference model for this purpose as an auditable framework that allows probability calibration and inspection of quantitative descriptors associated with disease-related image patterns. In this study a joint evaluation of a calibrated radiomics model and CNN baselines under the same data partition is proposed.

## 3 Materials and methods

### 3.1 Data sources and leakage-aware partitions

**Figure 1:**
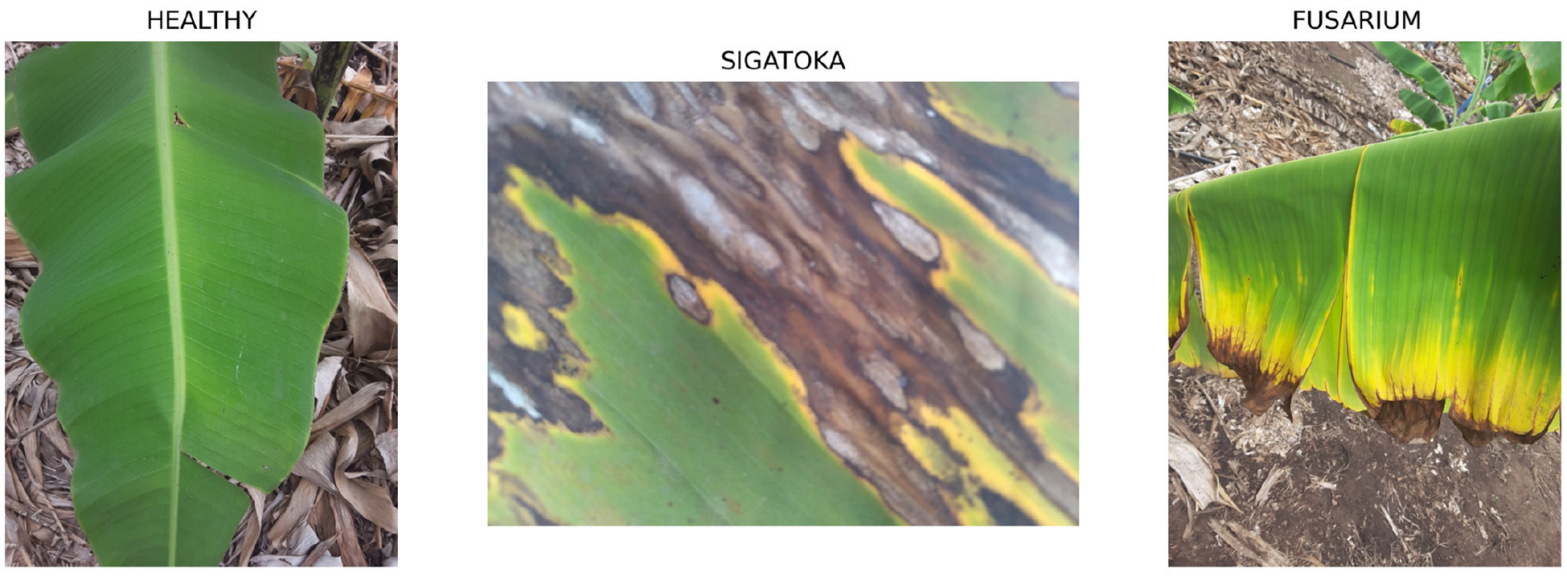
Representative banana leaf samples for the Healthy, Sigatoka, and Fusarium1 classes.

Two publicly available banana leaf image repositories were used to construct a three-class dataset comprising Healthy, Sigatoka, and Fusarium wilt race 1 (Fusarium1) samples. Images corresponding to Fusarium wilt and a subset of Healthy leaves were obtained from the Banana Leaves Imagery Dataset collected in Tanzania (Mduma et al., 2025; Arman et al., 2023). Additional Healthy samples and Sigatoka images were sourced from the Banana Leaf Disease Dataset Bangladesh (Arman et al., 2023). All images were RGB and organized in class-specific directories prior to preprocessing (Figure 1).

**Figure 2:**
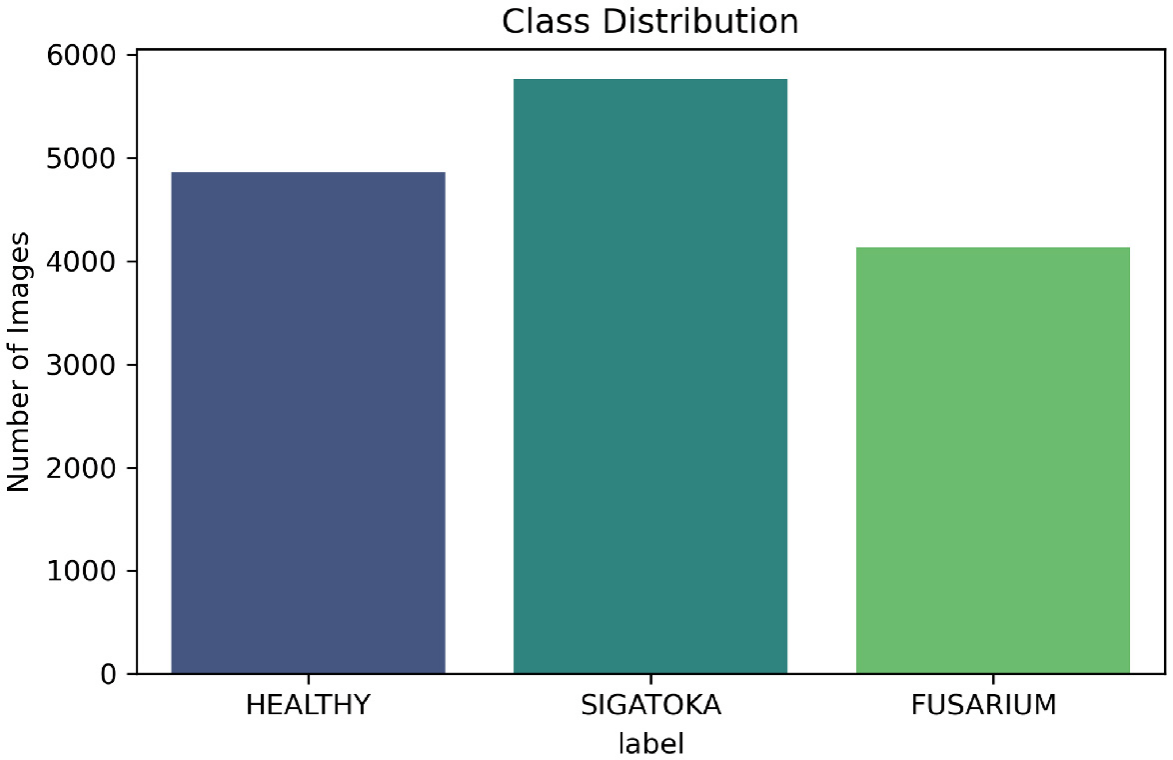
Dataset class distribution after radiomic feature extraction.

Following image segmentation and radiomic feature extraction, the resulting dataset consisted of *N* = 14, 763 radiomic samples. The class distribution after radiomic extraction was 4,866 samples for the Healthy class, 5,767 samples for the Sigatoka class, and 4,130 samples for the Fusarium1 class (Figure 2). This distribution reflects moderate class imbalance, motivating the use of balanced accuracy and macro-averaged F1-score throughout the study.

To reduce the risk of information leakage, the experimental protocol used a leakage-aware partitioning strategy based on original image identity. A group identifier was constructed from the original image path/stem, and samples derived from the same source image identity were assigned to only one of the training, validation, or test subsets. The dataset was partitioned into training, validation, and test subsets with proportions of 70%, 15%, and 15%, respectively. The test subset was held out and remained frozen during model selection, hyperparameter tuning, feature preprocessing, and probability calibration.

The public repositories used in this study do not provide verified plant-level, plot-level, or farm-level identifiers. Therefore, the present validation should not be interpreted as a plant-level or plot-level external validation. A stricter plant/plot-level or similarity-aware split, such as DataSAIL-like splitting (Joeres et al., 2025), would be preferable when the corresponding metadata or image-similarity matrices become available.

#### 3.1.1 Definition of experimental unit and radiomic samples

In this study, the experimental unit was defined at the image level. Each RGB image obtained from the public banana leaf repositories was associated with one class label according to the original dataset annotation: Healthy, Sigatoka, or Fusarium1. After HSV-based segmentation, a leaf-level region of interest (ROI) was extracted from each image and used for radiomic feature computation. Thus, each radiomic sample corresponds to one image-derived observation represented by a 103-dimensional radiomic feature vector.

The final dataset contained 14,763 radiomic samples, corresponding to 14,763 processed image-level observations. No patch-based subdivision, tiling, or multiple ROI extraction was performed; therefore, the analysis did not generate multiple radiomic vectors from the same image. The sample count refers to image-level radiomic observations rather than to patches or repeated crops extracted from the same image.

To reduce information leakage, the dataset partitioning was conducted using the original image identity before model training and evaluation. Consequently, image-derived observations sharing the same original image identity were assigned to only one subset: training, validation, or test. This procedure prevents the same source image from contributing derived samples to multiple partitions. However, because the public repositories do not provide verified plant-level, plot-level, or farm-level identifiers, the present split should be interpreted as leakage-aware at the image-identity level rather than as a plant-level or plot-level external validation. Stricter biological-unit splitting and similarity-aware partitioning remain necessary future steps when the corresponding metadata become available.

### 3.2 Image preprocessing and leaf segmentation

Prior to feature extraction, all RGB images underwent a standardized preprocessing pipeline designed to isolate leaf tissue from background elements such as soil, sky, shadows, and surrounding vegetation. Images were transformed from the RGB color space to HSV (Hue–Saturation–Value) space to exploit the partial separation between chromatic information and brightness. This representation was selected because it provides a transparent and computationally inexpensive way to define color-based leaf masks in the available RGB repositories. The HSV representation was obtained through the following color-space transformation (Equation 1). Let *I*_RGB_(*x, y*) denote the original RGB image at pixel location (*x, y*):


$$\begin{array}{l} I_{\text{HSV}}\left(x,y\right)=T_{\text{RGB}\to \text{HSV}}\left(I_{\text{RGB}}\left(x,y\right)\right), \end{array}$$ (1)

where *T*_RGB → HSV_(·) denotes the RGB-to-HSV transformation. The resulting HSV image contains three channels: hue *H*(*x, y*), saturation *S*(*x, y*), and value *V*(*x, y*). Hue represents the dominant chromatic component, saturation represents color purity, and value represents brightness.

In this study, leaf regions were identified using hue values in the range *H* ∈ [25°, 95°], corresponding to green and yellow-green tones typical of banana leaves under the evaluated acquisition conditions. To suppress low-chromaticity and dark background pixels, saturation and value thresholds were also applied, with *S* ≥ 20 and *V* ≥ 20. These values were selected experimentally to provide stable leaf-level segmentation for the available public repositories. However, fixed HSV thresholding should not be interpreted as universally robust under any illumination, weather, cultivar, canopy, or background conditions.

The binary leaf mask *M*(*x, y*) was defined as (Equation 2):


$$\begin{array}{l} M\left(x,y\right)=\{\begin{array}{ll} 1, & \text{ if }H_{\min}\leq H\left(x,y\right)\leq H_{\max},S\left(x,y\right)\geq S_{\min},V\left(x,y\right)\geq V_{\min},\\ 0, & \text{otherwise}, \end{array} \end{array}$$ (2)

where *H*_min_ = 25, *H*_max_ = 95, *S*_min_ = 20, and *V*_min_ = 20. The segmented image was then obtained by applying the binary mask to the original RGB image (Equation 3):


$$\begin{array}{l} I_{\text{ROI}}\left(x,y\right)=I_{\text{RGB}}\left(x,y\right)\cdot M\left(x,y\right), \end{array}$$ (3)

where *I*_ROI_ denotes the leaf-level region of interest used for subsequent radiomic feature extraction.

Morphological opening and closing operations were subsequently applied to remove isolated artifacts and improve spatial continuity of the segmented leaf region. Opening was used to reduce small foreground artifacts, whereas closing was used to fill small gaps inside the segmented mask. These operations can be expressed as (Equations 4, 5):


$$\begin{array}{l} M_{\text{open}}=\left(M⊖B\right)⊕B, \end{array}$$ (4)

$$\begin{array}{l} M_{\text{close}}=\left(M_{\text{open}}⊕B\right)⊖B, \end{array}$$ (5)

where ⊖ and ⊕ denote morphological erosion and dilation, respectively, and *B* denotes the structuring element. In this study, a 5 × 5 structuring element was used. When multiple connected components were present, the largest connected component was retained as the dominant leaf-level ROI to reduce background-induced variability.

**Figure 3:**
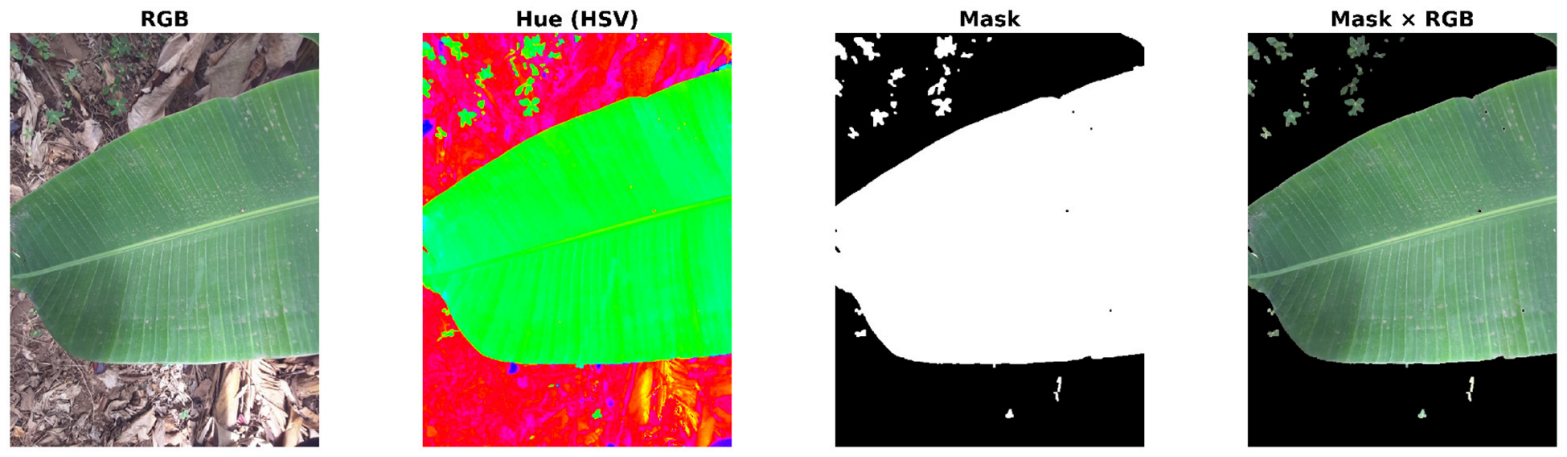
HSV-based leaf segmentation pipeline used to isolate banana leaf tissue from background elements. From left to right: original RGB image, HSV-derived representation, binary leaf mask obtained from thresholding, and final leaf-level ROI after morphological refinement.

Figure 3 illustrates the HSV-based segmentation pipeline, including the original RGB image, HSV-derived representation, binary leaf mask, and final segmented ROI after morphological refinement.

**Figure 4:**
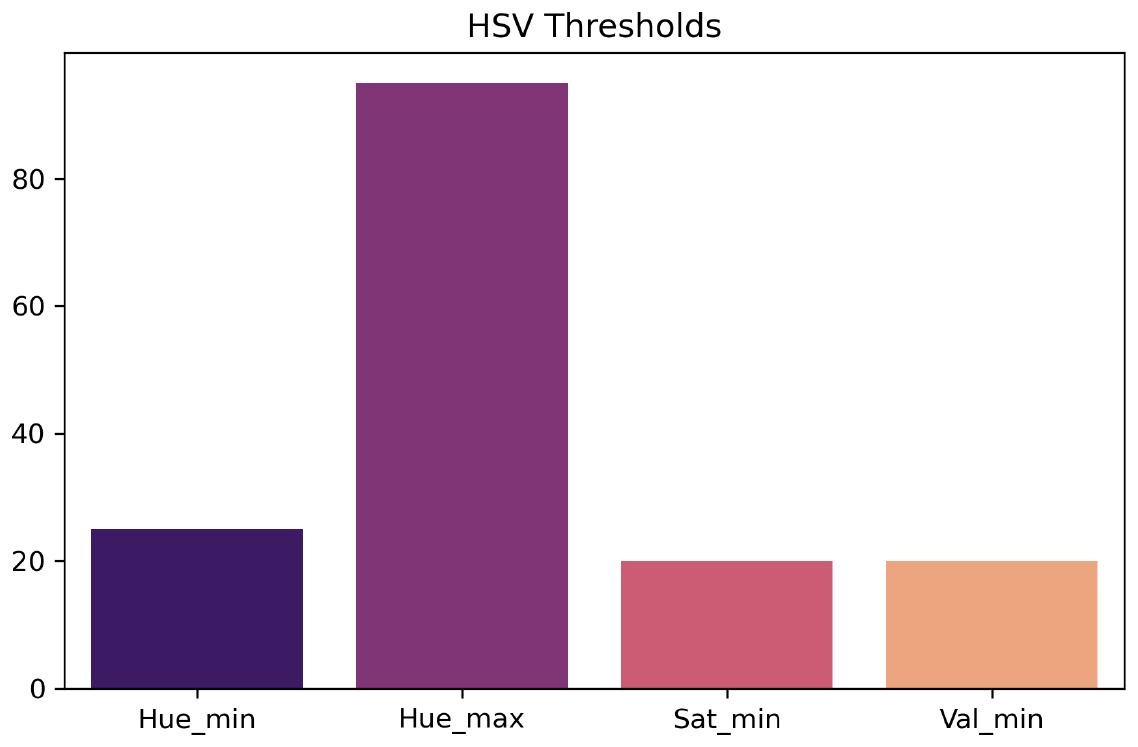
HSV threshold settings used for the leaf segmentation procedure. The selected interval *H* ∈ [25°, 95°], *S* ≥ 20, and *V* ≥ 20 was used to identify green and yellow-green banana leaf tissue in the evaluated repositories.

Figure 4 shows the HSV threshold settings used for the segmentation procedure.

To evaluate the sensitivity of the segmentation to the selected hue interval, an HSV sensitivity analysis was performed by perturbing the nominal hue range *H* ∈ [25°, 95°], while keeping the saturation and value thresholds fixed. Segmentation coverage was computed as (Equation 6):


$$\begin{array}{l} C=\frac{1}{\left|\Omega_{I}\right|}\underset{\left(x,y\right)\in \Omega_{I}}{\sum }M\left(x,y\right), \end{array}$$ (6)

where Ω_*I*_ denotes the image domain and *C* represents the proportion of pixels classified as leaf tissue. The mean coverage remained within a narrow range across the tested configurations, from 0.999398 to 0.999756, suggesting that the segmentation masks were stable for the evaluated repositories (Table 1). Nevertheless, this result should be interpreted as an internal sensitivity analysis rather than evidence of robustness under all uncontrolled field conditions.

**Table 1:** HSV sensitivity analysis of mean segmentation coverage under threshold perturbations.

Configuration	Mean coverage
Nominal *H* range [25°, 95°]	0.999756
Lower bound perturbed	0.999398
Upper bound perturbed	0.999621
Both bounds perturbed	0.999512

The narrow variation in mean segmentation of the selected HSV thresholds produced stable masks within the evaluated image repositories. However, field deployment would require additional validation under varying illumination, shadows, camera sensors, cultivar differences, canopy overlap, and background complexity.

### 3.3 Radiomic feature extraction

Fungal diseases affecting banana leaves induce a combination of localized necrosis, heterogeneous lesion expansion, discoloration, and progressive disruption of tissue organization. These pathological processes manifest in RGB images as variations in intensity distribution, spatial heterogeneity, textural irregularity, and alterations in the geometric structure of leaf regions. Radiomic texture matrices are well suited to characterize such image-level changes because they explicitly encode spatial relationships among pixel intensities at multiple scales and orientations, enabling quantitative differentiation between healthy tissue and disease-associated visual patterns. In contrast to end-to-end latent image representations, radiomic features provide explicitly defined descriptors that can later be inspected using feature-level interpretability methods.

Radiomic feature extraction was performed on the segmented leaf-level ROI obtained from the HSV-based segmentation pipeline. Each segmented ROI was converted into a grayscale image *I*(*x, y*) and discretized into a finite number of gray levels prior to feature computation. From this representation, multiple families of radiomic descriptors were computed to quantitatively characterize intensity distribution, shape, spatial organization, texture heterogeneity, and multiscale image structure associated with banana leaf disease patterns.

First-order statistics were computed directly from the intensity histogram *p*(*i*), where *i* ∈ {1, …, *G*} denotes the discretized gray levels and *G* is the total number of gray-level bins. These features include mean, variance, skewness, kurtosis, entropy, and intensity percentiles, and they describe the global distribution of pixel intensities within the segmented leaf region without considering spatial relationships. Shape descriptors were computed from the segmented ROI to characterize geometric properties of the leaf-level region, including measures related to size, perimeter, compactness, elongation, and sphericity. In this study, shape descriptors were retained because disease-associated tissue degradation, lesion expansion, and segmentation boundary irregularities can modify the geometry and regularity of the extracted ROI. These descriptors also provide interpretable quantities that can be inspected in subsequent permutation importance, SHAP, LIME, and ALE analyses.

Second-order texture features were derived from the gray-level co-occurrence matrix (GLCM), which encodes the joint probability of observing pairs of gray levels separated by a predefined spatial offset Δ = (Δ_*x*_, Δ_*y*_). The GLCM is defined as (Equation 7):


$$\begin{array}{l} P\left(i,j|\Delta \right)=\underset{x,y}{\sum }\{\begin{array}{ll} 1, & \text{ if }I\left(x,y\right)=i\text{ and }I\left(x+\Delta_{x},y+\Delta_{y}\right)=j,\\ 0, & \text{otherwise}, \end{array} \end{array}$$ (7)

and is normalized such that (Equation 8):


$$\begin{array}{l} \overset{G}{\underset{i=1}{\sum }}\overset{G}{\underset{j=1}{\sum }}P\left(i,j|\Delta \right)=1. \end{array}$$ (8)

From the GLCM, features such as contrast, correlation, homogeneity, energy, and entropy were extracted to capture local spatial dependencies and texture regularity associated with visible disease-related patterns.

Higher-order texture characterization was achieved using the gray-level run-length matrix (GLRLM), which quantifies the length of consecutive runs of pixels with the same gray level along a specified direction. The GLRLM *R*(*i, r*) represents the number of runs with gray level *i* and run length *r*. A run is defined as a sequence of *r* contiguous pixels having the same gray level *i*. Features derived from the GLRLM, including short-run emphasis, long-run emphasis, gray-level non-uniformity, and run-length non-uniformity, quantify directional texture continuity and anisotropy. These properties may be relevant for elongated lesions, streak-like symptoms, or non-uniform tissue alterations.

Complementary spatial heterogeneity information was also extracted using the gray-level size-zone matrix (GLSZM), which characterizes the size of homogeneous zones irrespective of orientation. A zone is defined as a connected region of pixels with identical gray level *i*. The GLSZM *Z*(*i, s*) denotes the number of zones with gray level *i* and size *s*. Features derived from the GLSZM, such as small-area emphasis, large-area emphasis, zone-size non-uniformity, and gray-level non-uniformity, capture patchy lesion distributions and irregular spatial organization within the segmented leaf region.

Local intensity dependency was further modeled using the gray-level dependence matrix (GLDM). A dependence is defined as a set of neighboring pixels within a predefined distance δ whose gray-level difference relative to a center pixel does not exceed a given threshold. The GLDM *D*(*i, d*) represents the number of pixels with gray level *i* that have a dependence size *d*. Features derived from the GLDM describe the strength and uniformity of local dependencies, providing sensitivity to local texture disruption and tissue heterogeneity.

Additionally, perceptual texture characteristics were captured using the neighboring gray-tone difference matrix (NGTDM). For each gray level *i*, the NGTDM computes the difference between the intensity of pixels with gray level *i* and the average gray level of their neighboring pixels. The NGTDM element *N*(*i*) is defined as (Equation 9):


$$\begin{array}{l} N\left(i\right)=\underset{k\in P_{i}}{\sum }|i-{\bar{I}}_{N\left(k\right)}|, \end{array}$$ (9)

where *P*_*i*_ denotes the set of pixels with gray level *i*, and ${\bar{I}}_{N\left(k\right)}$ is the mean gray level of the neighborhood of pixel *k*. Derived features, including coarseness, contrast, busyness, complexity, and texture strength, quantify perceptual smoothness and local variability, which may be altered by fungal lesion development and tissue degradation.

To capture disease-related patterns at multiple spatial scales, discrete wavelet transforms were applied to the segmented leaf images prior to feature extraction. Radiomic features from the families described above were computed on both the original and wavelet-filtered representations. This multiresolution strategy provides sensitivity to both fine-grained texture changes and broader structural alterations.

The complete feature extraction process yielded one radiomic feature vector per segmented image-level ROI (Equation 10):


$$\begin{array}{l} {\text{x}}_{n}=\left[x_{n,1},x_{n,2},\ldots ,x_{n,d}\right]\in {\mathbb{R}}^{d},\;d=103, \end{array}$$ (10)

where **x**_*n*_ denotes the radiomic representation of image-level sample *n*. These 103 descriptors were computed before feature curation. Subsequent preprocessing, including missing-value imputation, variance filtering, standardization, and Pearson correlation filtering, was fitted using the training subset only, as described in Section 3.4.

**Figure 5:**
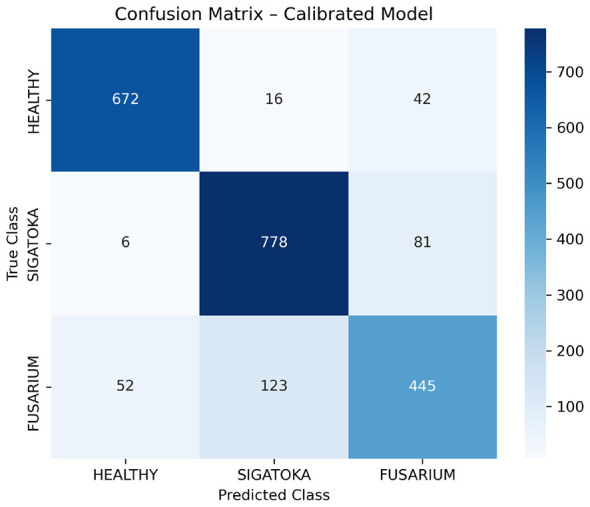
Overview of the radiomics-based feature extraction workflow. Raw RGB images undergo HSV-based leaf segmentation to obtain a leaf-level region of interest (ROI), followed by radiomic feature computation, yielding a quantitative vector of shape, first-order, texture, and wavelet-based descriptors.

An overview of the radiomics-based feature extraction pipeline is provided in Figure 5.

### 3.4 Feature preprocessing

Prior to model training, feature preprocessing was applied in a training-only manner to avoid information leakage. Missing values were first analyzed across the radiomic feature table. Although radiomic feature extraction is expected to generate a descriptor vector for each segmented ROI, missing or non-finite values may occur in practice due to segmentation artifacts, very small or nearly homogeneous ROIs, empty texture matrices, or numerical instability in specific feature computations. The radiomics extraction pipeline was configured to minimize missing and non-finite values. Nevertheless, a predefined quality-control rule was included to ensure robustness and reproducibility: descriptors with more than 20% missing or non-finite values in the training subset would be excluded before model fitting. In the present dataset, no descriptor met this exclusion criterion; therefore, the reported results were not affected by feature removal due to missingness. Any remaining missing or non-finite entries, if present, were imputed using medians estimated exclusively from the training subset and then applied unchanged to the validation and test subsets.

Features were standardized using *z*-score normalization, defined as (Equation 11):


$$\begin{array}{l} x_{n,j}^{\prime }=\frac{x_{n,j}-\mu_{j}^{\text{train}}}{\sigma_{j}^{\text{train}}}, \end{array}$$ (11)

where *x*_*n,j*_ denotes the value of feature *j* for sample *n*, and $\mu_{j}^{\text{train}}$ and $\sigma_{j}^{\text{train}}$ denote the mean and standard deviation of feature *j* computed only on the training subset. This transformation ensured that validation and test samples were scaled using parameters estimated without access to held-out data.

To reduce redundancy and improve model stability, near-constant descriptors were removed using a variance threshold of 10^−6^. Highly correlated features were then identified using Pearson correlation analysis fitted only on the training data. For each pair of descriptors with absolute correlation greater than 0.95, one feature was removed. In the list of radiomic features, 102 numeric descriptors were available after excluding class labels and image-path metadata. No descriptor was removed by the missing-value threshold or the variance threshold; Pearson correlation filtering removed 58 highly correlated descriptors, leaving 44 features for downstream modeling. The same preprocessing parameters were applied unchanged to the validation and test subsets. Table 2 summarizes the preprocessing steps and their results.

**Table 2:** Feature preprocessing summary.

Step	Criterion	Outcome
Metadata exclusion	label and img_path fields removed	102 numeric radiomic descriptors available
Missing-value filtering	Features with more than 20% missing values removed	0 descriptors removed; 102 descriptors retained
Imputation	Training-set median	Remaining missing values imputed using training-set statistics
Variance filtering	Threshold = 10^−6^, fitted on training data only	0 descriptors removed; 102 descriptors retained
Pearson correlation filtering	Pairwise absolute Pearson correlation fitted on training data only; for each pair with absolute correlation greater than 0.95, one descriptor was removed	58 highly correlated descriptors removed; 44 descriptors retained for downstream modeling
Standardization	*z*-score normalization using training-set mean and standard deviation	The 44 retained descriptors were standardized and used as inputs to the radiomics classifiers

### 3.5 Machine learning models and training procedure

Four supervised radiomics-based classifiers were evaluated: Logistic Regression with *L*_2_ regularization, a Ridge classifier, a Support Vector Machine (SVM) with radial basis function kernel, and a Random Forest. Training was performed using the training subset, while model selection and hyperparameter selection relied on validation performance. The held-out test subset remained frozen and was used only for final evaluation.

The Random Forest model was trained using balanced class weights to compensate for moderate class imbalance. Class weights were computed inversely proportional to class frequencies in the training data, so that minority classes contributed proportionally more to the training objective. The Random Forest was selected as the final classical radiomics model because it achieved the strongest performance among the evaluated radiomics classifiers.

Model performance was evaluated using accuracy, balanced accuracy, macro-averaged F1-score, and macro-averaged one-vs-rest area under the ROC curve. Balanced accuracy was computed as (Equation 12):


$$\begin{array}{l} \text{BA}=\frac{1}{C}\overset{C}{\underset{c=1}{\sum }}\frac{{\text{TP}}_{c}}{{\text{TP}}_{c}+{\text{FN}}_{c}}, \end{array}$$ (12)

where *C* denotes the number of classes, and TP_*c*_ and FN_*c*_ denote true positives and false negatives for class *c*, respectively.

In addition to overall accuracy and balanced accuracy, class-wise precision, recall, and F1-score were computed to provide a detailed evaluation of classification performance under class imbalance. For each class *c*, precision and recall are defined as (Equations 13):


$$\begin{array}{l} {\text{Precision}}_{c}=\frac{{\text{TP}}_{c}}{{\text{TP}}_{c}+{\text{FP}}_{c}},\;{\text{Recall}}_{c}=\frac{{\text{TP}}_{c}}{{\text{TP}}_{c}+{\text{FN}}_{c}}, \end{array}$$ (13)

where FP_*c*_ denotes false positives for class *c*. The class-wise F1-score was computed as (Equation 14):


$$\begin{array}{l} {\text{F1}}_{c}=2\cdot \frac{{\text{Precision}}_{c}\cdot {\text{Recall}}_{c}}{{\text{Precision}}_{c}+{\text{Recall}}_{c}}. \end{array}$$ (14)

The macro-averaged F1-score was then defined as (Equation 15):


$$\begin{array}{l} {\text{F1}}_{\text{macro}}=\frac{1}{C}\overset{C}{\underset{c=1}{\sum }}{\text{F1}}_{c}. \end{array}$$ (15)

This metric assigns equal weight to each class regardless of class frequency, making it suitable for the moderately imbalanced multiclass setting considered in this study.

The comparative performance of the four radiomics-based classifiers is reported in the Results section to support the selection of the Random Forest as the final calibrated radiomics model.

### 3.6 CNN baselines

Three CNN architectures were included as baselines: MobileNetV3, EfficientNet, and ResNet-18. These families of CNN architectures were selected because they have been widely used for image classification and provide appropriate image-based benchmarks for comparison with the radiomics pipeline.

The CNN models were trained and evaluated using the same train/validation/test partition used for the radiomics experiments. The comparison between radiomics and CNN models was conducted under the same data partition and class distribution. RGB images were resized to 224 × 224 pixels and normalized using ImageNet mean and standard deviation values. The final classification layer of each architecture was replaced with a three-class output layer corresponding to Healthy, Sigatoka, and Fusarium1.

These models were initialized with ImageNet-pretrained weights and fine-tuned using cross-entropy loss. For a sample *n* with true class label *y*_*n*_ and predicted probability ${\hat{p}}_{n,c}$ for class *c*, the multiclass cross-entropy loss was defined as (Equation 16):


$$\begin{array}{l} L_{\text{CE}}=-\frac{1}{N}\overset{N}{\underset{n=1}{\sum }}\overset{C}{\underset{c=1}{\sum }}𝟙\left(y_{n}=c\right)\log\left({\hat{p}}_{n,c}\right), \end{array}$$ (16)

where *N* is the number of training samples, *C* = 3 is the number of classes, and 𝟙(·) is the indicator function. Optimization was performed using Adam. Model selection was based on validation macro-F1, and the best checkpoint was evaluated on the held-out test set.

The purpose of these CNN baselines was to establish a performance image-based benchmark. This allows the study to quantify the difference between raw predictive performance and feature-level interpretability under the same data partition.

### 3.7 Probability calibration and interpretability analysis

Post-hoc probability calibration was applied to the selected Random Forest classifier because reliable confidence scores are required for risk-aware decision support. Since probability calibration does not alter class predictions but adjusts the reliability of predicted confidence scores, calibration was restricted to the final selected radiomics model intended for interpretability and decision-support analysis.

Ensemble-based classifiers such as Random Forest may produce poorly calibrated probability estimates because class probabilities are obtained by averaging discrete decision-tree outputs and can be biased by finite leaf sample sizes (Dankowski and Ziegler, 2016). This characteristic motivates the use of post-hoc calibration techniques when reliable probability estimates are required, as in phytosanitary surveillance and risk-aware agricultural decision support.

Probability calibration was performed using Isotonic Regression, a non-parametric monotonic calibration method that learns a function mapping raw model scores to calibrated probabilities, which is defined as follows. Let *s*_*i*_ ∈ [0, 1] denote the uncalibrated probability output of the Random Forest model for sample *i*, and let *y*_*i*_ ∈ {0, 1} represent the corresponding binary indicator for a given class in a one-vs-rest setting. Isotonic regression estimates a non-decreasing function *f*(·) by solving (Equation 17):


$$\begin{array}{l} \underset{f}{\min}\;\overset{N}{\underset{i=1}{\sum }}{\left(f\left(s_{i}\right)-y_{i}\right)}^{2}\text{ s.t. }f\left(s_{i}\right)\leq f\left(s_{j}\right)\text{ whenever }s_{i}\leq s_{j}. \end{array}$$ (17)

The calibrated probability for each class is then given by (Equation 18):


$$\begin{array}{l} {\hat{p}}_{i}=f\left(s_{i}\right). \end{array}$$ (18)

Calibration was fitted without using the held-out test set, and the learned calibration function was subsequently applied to the test set. Calibration quality was assessed using class-wise reliability diagrams and Brier score decomposition. Calibration was interpreted as a mechanism to improve confidence reliability, not as a method to improve biological validity or change categorical predictions.

Following probability calibration, interpretability analysis was conducted on the calibrated Random Forest model to ensure that explanations reflected the behavior of the final radiomics predictive system. Given the high-dimensional nature of the radiomic feature space, a combination of global and local explainability techniques was employed.

Global feature relevance was assessed using permutation-based feature importance, which measures the decrease in predictive performance after randomly shuffling individual features. SHAP values were computed to characterize feature contributions using a game-theoretic attribution framework (Lundberg and Lee, 2017). For each feature *j*, the SHAP value ϕ_*j*_ represents its marginal contribution to the model prediction across coalitions of features, enabling consistent feature-level attribution.

Local interpretability was addressed using LIME (Ribeiro et al., 2016), which approximates the Random Forest decision function locally by fitting an interpretable surrogate model around individual predictions. Finally, ALE analysis was used to inspect non-linear associations between selected radiomic descriptors and predicted class probabilities (Molnar, 2023). ALE was interpreted as exploratory evidence of non-linear feature effects, not as proof of specific feature interactions.

### 3.8 Statistical analysis

To quantify uncertainty in model performance, non-parametric bootstrap confidence intervals were computed for the main Random Forest metrics on the held-out test set. Bootstrap resampling was performed at the sample level by repeatedly drawing test samples with replacement and recomputing the evaluation metric for each bootstrap replicate. For a metric *m*, the empirical bootstrap distribution was defined as (Equation 19):


$$\begin{array}{l} \left\{m^{*\left(1\right)},m^{*\left(2\right)},\ldots ,m^{*\left(B\right)}\right\}, \end{array}$$ (19)

where *B* denotes the number of bootstrap replicates. The 95% confidence interval was estimated using the 2.5th and 97.5th percentiles of this empirical distribution (Equation 20):


$$\begin{array}{l} {\text{CI}}_{95\%}\left(m\right)=\left[Q_{0.025}\left(m^{*}\right),Q_{0.975}\left(m^{*}\right)\right]. \end{array}$$ (20)

Paired classifier comparison was performed using McNemar testing on predictions from the same held-out test samples. McNemar testing was used to assess whether two classifiers differed in their paired error patterns. For two classifiers A and B, let *n*_01_ denote the number of samples misclassified by classifier A but correctly classified by classifier B, and let *n*_10_ denote the number of samples correctly classified by classifier A but misclassified by classifier B. The McNemar statistic was computed as (Equation 21):


$$\begin{array}{l} \chi^{2}=\frac{{\left(n_{01}-n_{10}\right)}^{2}}{n_{01}+n_{10}}. \end{array}$$ (21)

This test allowed the comparison of the Random Forest and SVC-RBF predictions on the same held-out test set. The resulting statistic and *p*-value are reported in the Results section. These statistical analyses complemented the point estimates and provided an assessment of model uncertainty and paired prediction differences under the evaluated data partition.

## 4 Results

After radiomic feature extraction, the final dataset used for the model development and evaluation consisted of *N* = 14, 763 radiomic samples represented by 103 radiomic descriptors prior to feature curation. The class distribution comprised 4,866 Healthy samples, 5,767 Sigatoka samples, and 4,130 Fusarium1 samples. The moderate class imbalance motivated the use of balanced accuracy and macro-F1 as primary evaluation metrics.

Segmentation was performed using the HSV-based pipeline described in Section 3.2. The HSV sensitivity analysis showed that the mean segmentation coverage remained within a narrow range across the tested threshold perturbations, from 0.999398 to 0.999756. The selected HSV thresholds produced stable masks within the evaluated repositories.

### 4.1 Comparative performance of radiomics-based classifiers

Four radiomics-based classifiers were evaluated using the leakage-aware experimental protocol described in Section 3. Table 3 reports the comparative performance of the Random Forest, Ridge classifier, Logistic Regression with *L*_2_ regularization, and RBF-kernel SVC on the held-out test set.

**Table 3:** Comparative performance of radiomics-based classifiers on the held-out test set.

Model	Accuracy	Balanced accuracy	Macro-F1
Random Forest	0.8447	0.8319	0.8345
Ridge classifier	0.7377	0.7110	0.6991
Logistic Regression (*L*_2_)	0.6474	0.6174	0.6050
SVC-RBF	0.6266	0.5814	0.5292

The Random Forest achieved the strongest performance among the evaluated radiomics classifiers. As shown in Table 4, the refined and calibrated Random Forest achieved an accuracy = 0.8555, balanced accuracy = 0.8459, macro-F1 = 0.8476, and macro ROC-AUC OvR = 0.9576 on the held-out test set. The bootstrap analysis yielded a 95% confidence interval of [0.8406, 0.8691] for accuracy and [0.8306, 0.8606] for balanced accuracy. The McNemar testing showed a statistically significant difference between the paired error patterns of Random Forest and SVC-RBF on the same held-out test set, with statistic = 364.2048 and *p* = 3.420 × 10^−81^.

**Table 4:** Final calibrated Random Forest performance and statistical uncertainty.

Metric	Value	95% bootstrap CI/ test
Accuracy	0.85	[0.8406, 0.8691]
Balanced accuracy	0.84	[0.8306, 0.8606]
Macro-F1	0.84	Not applicable
Macro ROC-AUC OvR	0.95	Not applicable
McNemar RF vs. SVC-RBF	Statistic = 364.2048	*p* = 3.420 × 10^−81^

The class-wise analysis showed that the calibrated Random Forest performed differently across the three categories. A detailed summary of per-class classification performance for the best calibrated model is reported in Table 5.

**Table 5:** Per-class performance of the Random Forest radiomics model on the held-out test set.

Class	Precision	Recall	F1-score	Support
Healthy	0.9317	0.9151	0.9233	730
Sigatoka	0.8279	0.9064	0.8653	865
Fusarium1	0.7840	0.6968	0.7378	620

**Figure 6:**
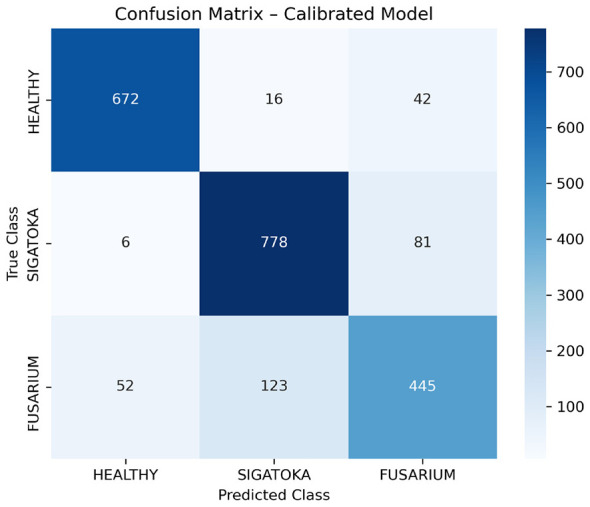
Confusion matrix of the calibrated Random Forest classifier on the test set.

The confusion matrix in Figure 6 shows that a subset of Fusarium1 samples was confused with Sigatoka. This error type has operational implications because Fusarium wilt, particularly Foc R4T, is soilborne and requires quarantine, sanitation, movement restriction, and confirmatory laboratory testing. Therefore, radiomics predictions should be interpreted as decision-support triage outputs rather than direct treatment recommendations.

Also, while false positives may increase inspection workload and generate unnecessary concern, false negatives could delay field inspection, containment measures, sanitation, movement restrictions, and laboratory confirmation. Therefore, low-confidence predictions, ambiguous classifications, or any prediction involving Fusarium-like symptoms should trigger expert inspection and confirmatory testing.

### 4.2 Comparison with CNN baselines

To quantify the accuracy–interpretability trade-off, three CNN baselines were trained and evaluated on the same data partition: MobileNetV3, EfficientNet, and ResNet-18. Table 6 compares these CNN baselines with the calibrated radiomics Random Forest.

**Table 6:** Comparative performance of the calibrated radiomics model and CNN baselines on the same held-out test set.

Model/input	Accuracy	Balanced acc.	Macro-F1	ROC-AUC OvR
Radiomics RF	0.85	0.84	0.84	0.95
MobileNetV3	0.96	0.96	0.95	0.97
EfficientNet	0.96	0.96	0.97	0.96
ResNet-18	0.96	0.96	0.96	0.96

All three CNN baselines achieved better performance on the evaluated repositories. EfficientNet and ResNet-18 obtained the highest overall accuracy = 0.96, with macro-F1 values of 0.97 and 0.96, respectively, while MobileNetV3 achieved an accuracy = 0.96 and macro-F1 = 0.95. The differences among the three CNNs showed that the evaluated repositories contain separable image-level patterns that can be captured by multiple compact architectures. The radiomics-based model provides a calibrated, auditable, and feature-level interpretable reference model that complements the performing image-based CNNs.

### 4.3 Probability calibration of the radiomics model

**Figure 7:**
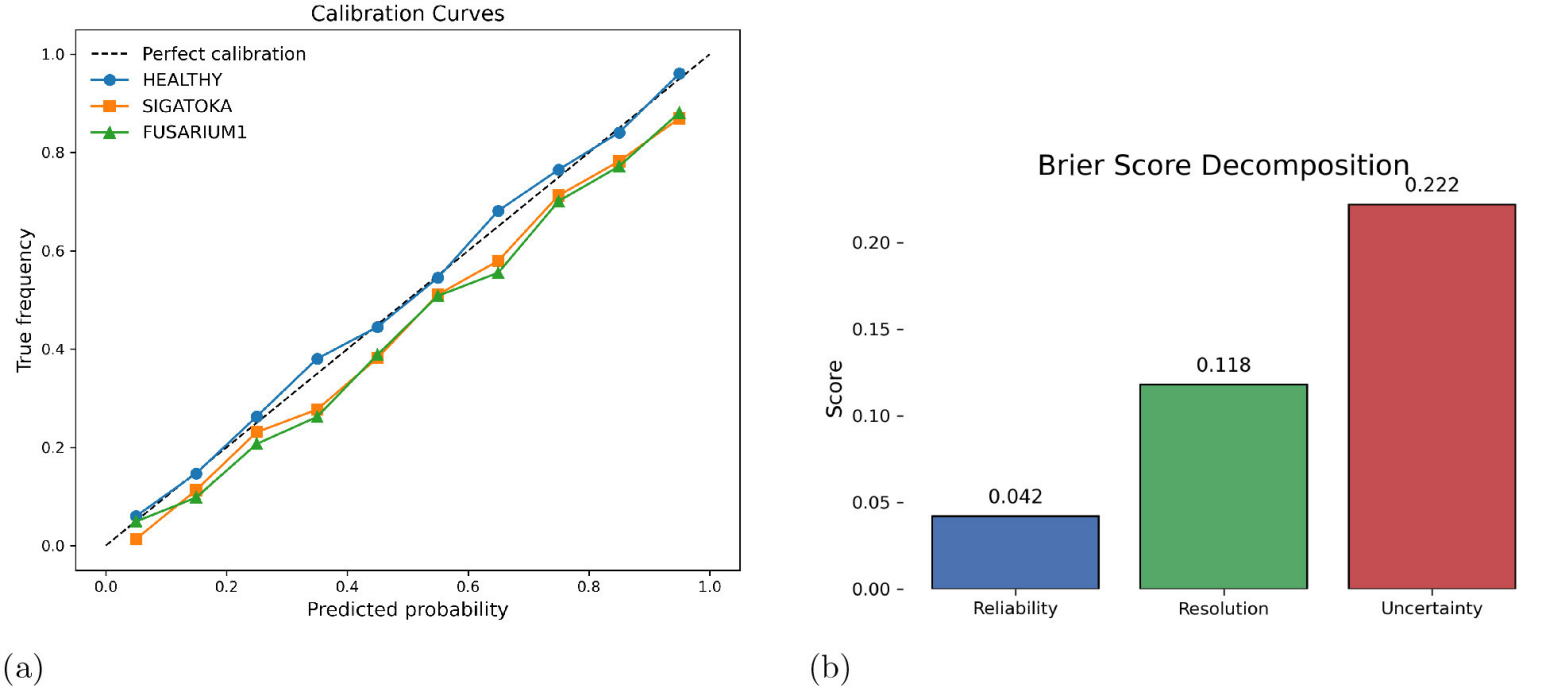
Probability calibration assessment of the calibrated Random Forest classifier. **(a)** Class-wise reliability diagrams after isotonic regression. **(b)** Brier score decomposition into reliability, resolution, and uncertainty components.

Besides the categorical performance, probability calibration was also assessed for the selected Random Forest model. The reliability diagrams in Figure 7 show the relationship between predicted probabilities and observed class frequencies after isotonic calibration. The calibrated curves followed the diagonal reference line reasonably closely, indicating improved agreement between predicted confidence and empirical outcomes. The Brier score decomposition in Figure 7 also showed a low reliability component relative to uncertainty, supporting the use of calibrated probabilities for risk-aware triage and threshold-based inspection workflows.

These calibration results provided an improved confidence reliability under the evaluated test distribution.

### 4.4 Global feature relevance: permutation importance and SHAP

**Figure 8:**
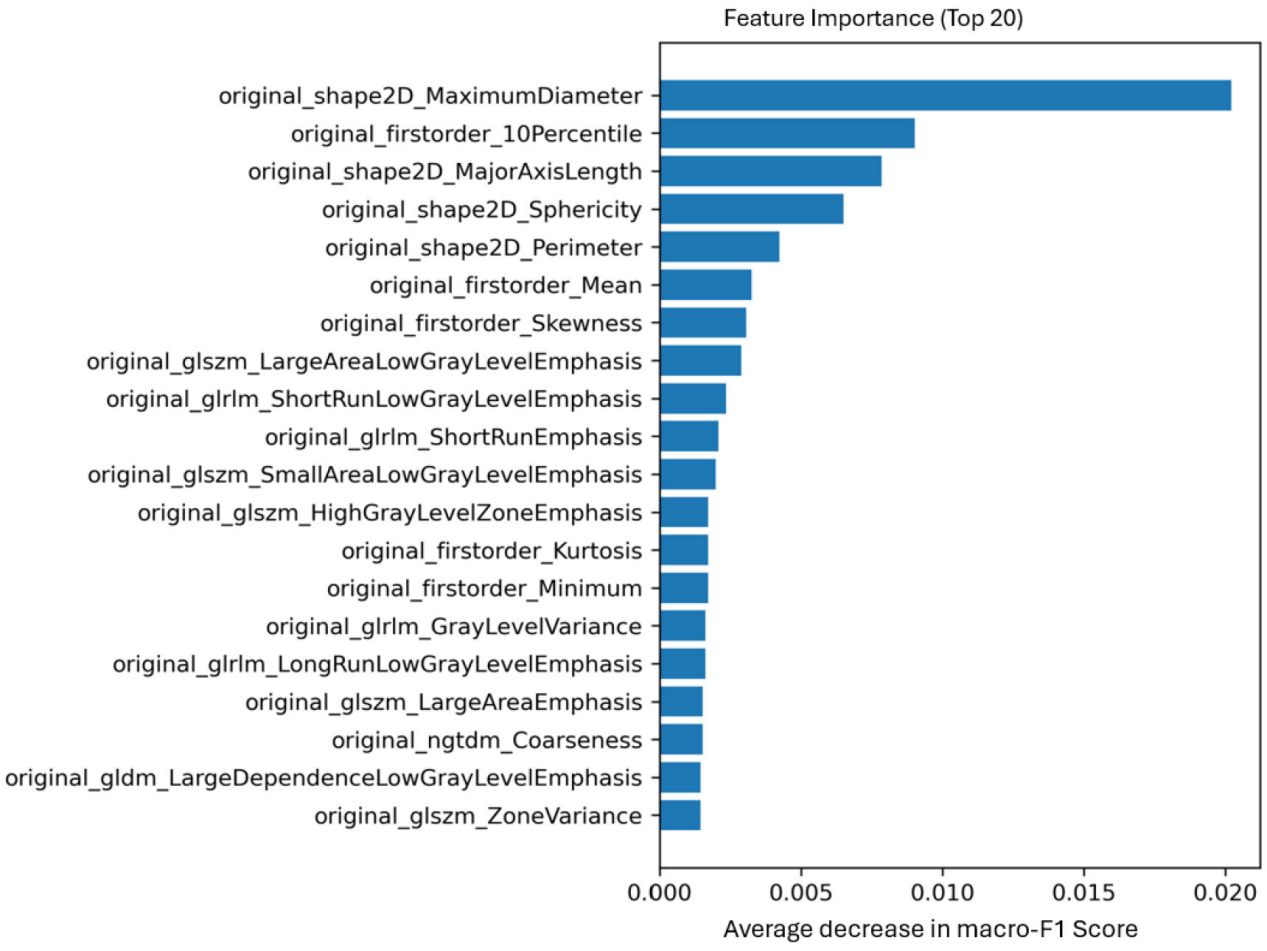
Permutation-based feature importance for the calibrated Random Forest classifier.

Permutation-based feature importance was used to evaluate the global relevance of individual radiomic descriptors. Figure 8 shows that the largest decrease in macro-F1 occurred when original_shape2D_

MaximumDiameter was permuted; this descriptor had the strongest global contribution to the calibrated Random Forest. Other highly ranked features included original_firstorder_

10Percentile, original_shape2D_

MajorAxisLength, original_shape2D_

Sphericity, original_shape2D_

Perimeter, original_firstorder_

Mean, and original_firstorder_

Skewness. Texture descriptors from GLSZM and GLRLM families also appeared among the top-ranked features, including original_glszm_

LargeAreaLowGrayLevelEmphasis, original_glrlm_

ShortRunLowGrayLevelEmphasis, and original_glrlm_

ShortRunEmphasis.

The permutation importance results indicate that the radiomics model relied primarily on shape, intensity-distribution, and texture-heterogeneity descriptors. This is consistent with the objective of the radiomics framework: to provide feature-level evidence that can be inspected as quantitative image traits rather than latent CNN activations.

**Figure 9:**
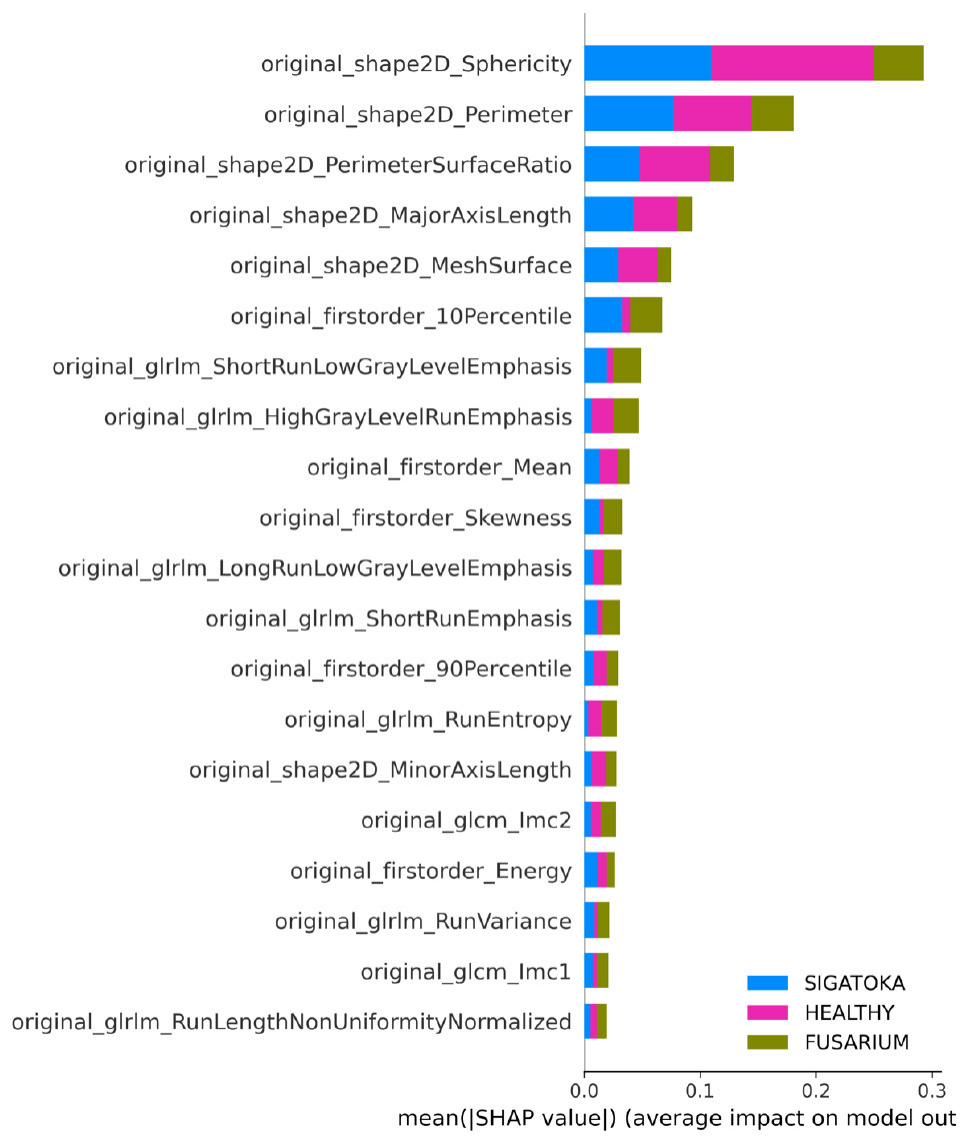
Global SHAP summary plot across all classes.

The global SHAP summary in Figure 9 shows a similar pattern. Across classes, original_shape2D_

Sphericity had the highest mean absolute SHAP contribution, followed by original_shape2D_

Perimeter, original_shape2D_

PerimeterSurfaceRatio, original_shape2D_

MajorAxisLength, and original_shape2D_

MeshSurface. First-order intensity descriptors and run-length texture descriptors also contributed to the model output. The class-stacked SHAP bars further show that the contribution of each descriptor was not uniform across classes; shape descriptors contributed strongly to Healthy and Sigatoka predictions, whereas several texture descriptors contributed to disease-related predictions, including Fusarium1.

### 4.5 Class-wise SHAP interpretation

Class-wise SHAP analyses were used to examine how feature values influenced the model output for each class.

**Figure 10:**
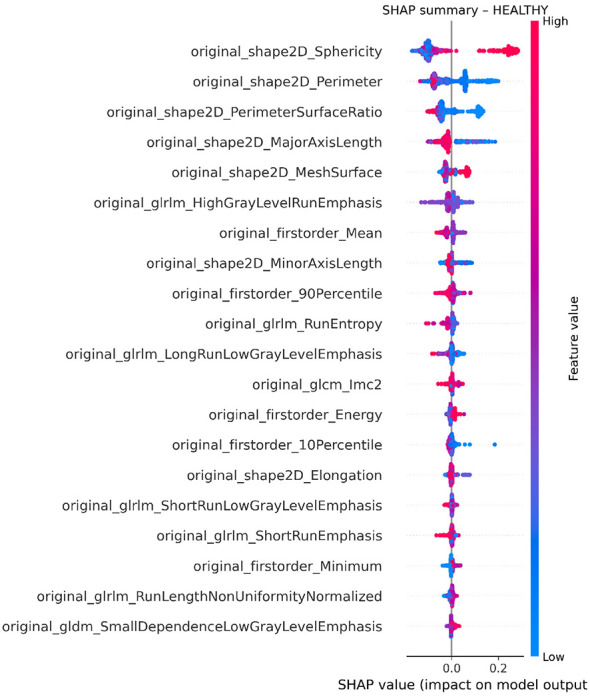
SHAP summary plot for the Healthy class.

For the Healthy class, Figure 10 shows that original_shape2D_

Sphericity was the dominant feature. Higher sphericity values tended to increase the model output for Healthy, whereas lower or intermediate values in perimeter-related descriptors contributed differently across samples. The Healthy explanation was also influenced by original_shape2D_

Perimeter, original_shape2D_

PerimeterSurfaceRatio, original_shape2D_

MajorAxisLength, and original_shape2D_

MeshSurface, showing that predictions for healthy tissue were mainly driven by geometric regularity and shape-related descriptors. Texture features, such as original_glrlm_

HighGrayLevelRunEmphasis, and first-order statistics contributed less strongly but still appeared among the relevant descriptors.

**Figure 11:**
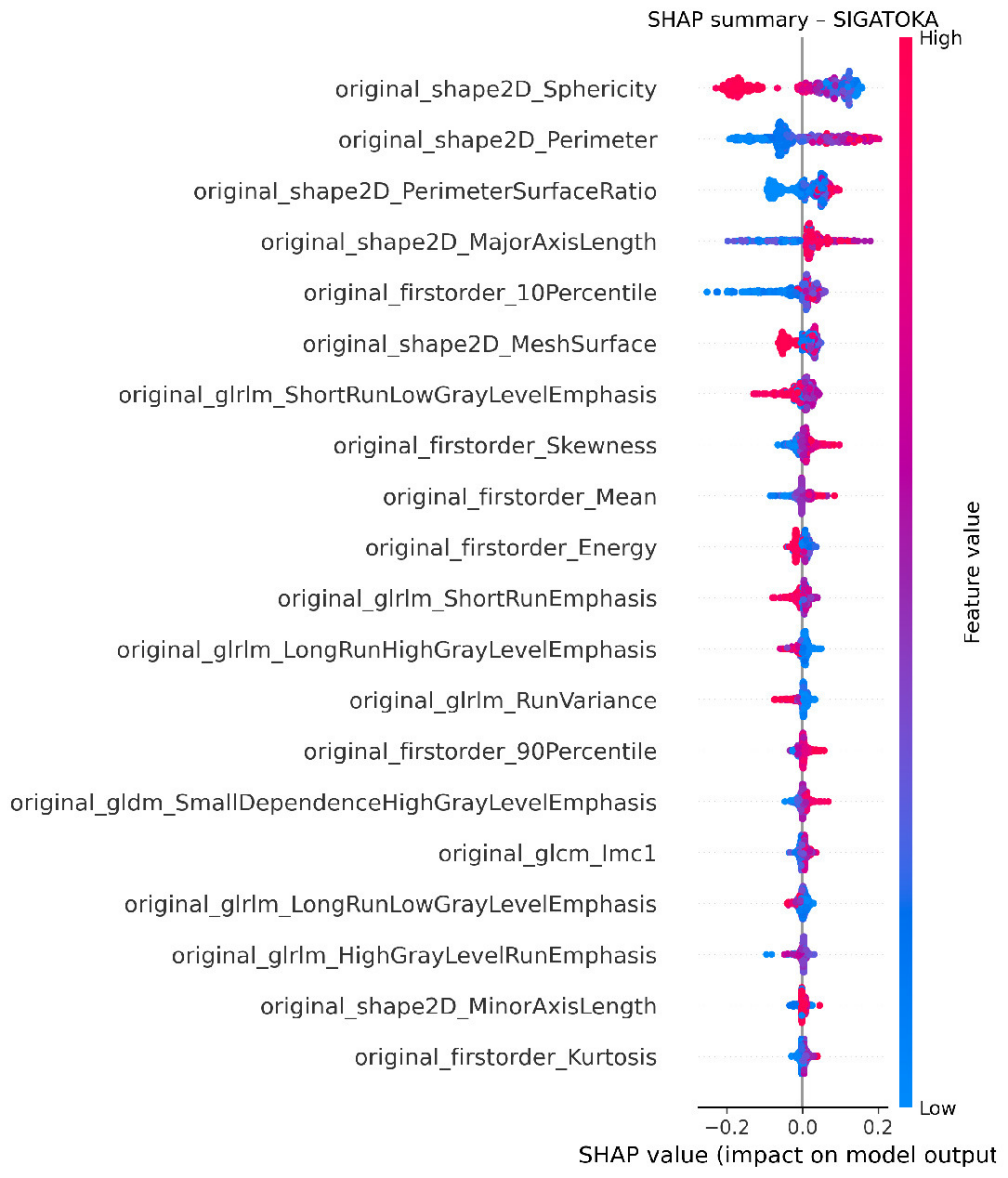
SHAP summary plot for the Sigatoka class.

For the Sigatoka class, Figure 11 shows that lower values of original_shape2D_

Sphericity tended to increase the Sigatoka model output, while higher values tended to reduce it. Conversely, higher values of perimeter-related and elongation-related features, including original_shape2D_

Perimeter, original_shape2D_

PerimeterSurfaceRatio, and original_shape2D_

MajorAxisLength, contributed positively to Sigatoka predictions. The Random Forest associated Sigatoka with less regular shape patterns and more extended or irregular segmented regions. First-order intensity features and run-length descriptors provided additional contributions; both shape and texture influenced Sigatoka classification.

**Figure 12:**
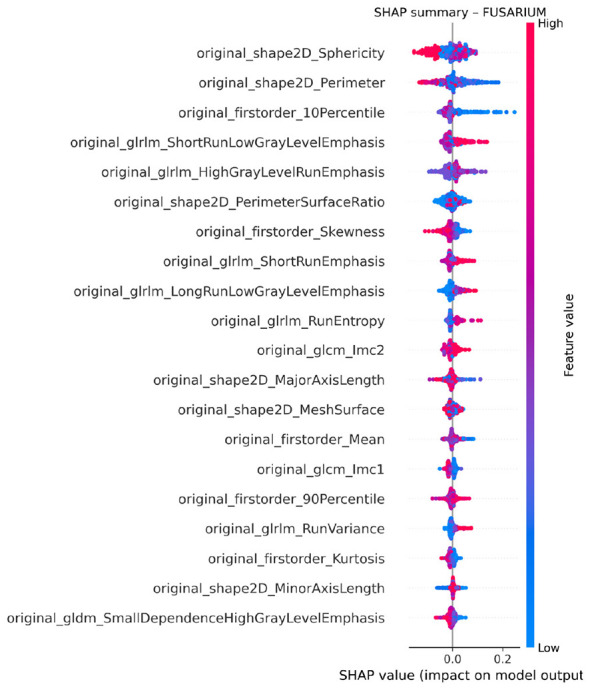
SHAP summary plot for the Fusarium1 class.

For the Fusarium1 class, Figure 12 shows a different attribution pattern. Shape descriptors, such as original_shape2D_

Sphericity, original_shape2D_

Perimeter, and original_shape2D_

PerimeterSurfaceRatio, remained relevant, but texture descriptors had a more visible role. Features such as original_glrlm_

ShortRunLowGrayLevelEmphasis, original_glrlm_

HighGrayLevelRunEmphasis, original_glrlm_

ShortRunEmphasis, original_glrlm_

RunEntropy, original_glcm_

Imc2, and original_glrlm_

RunVariance contributed to the model output. The Fusarium1 predictions relied not only on shape regularity but also on texture patterns associated with local gray-level variation and run-length structure.

These explanations provide traceable quantitative descriptors that can be inspected by researchers and agronomists. Unlike the CNN feature maps, these descriptors are directly associated with interpretable measurements such as perimeter, sphericity, intensity percentiles, and texture non-uniformity.

### 4.6 Local explanations using LIME

LIME was used to provide sample-level explanations for representative test predictions. These local explanations complement the global SHAP analysis by showing how specific feature intervals influenced individual predictions.

**Figure 13:**
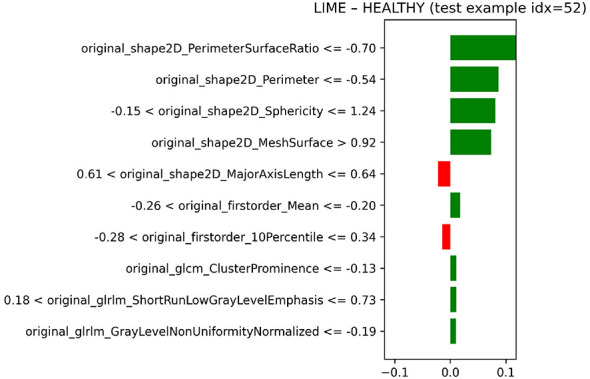
LIME explanation for a representative correctly classified Healthy sample.

For a representative Healthy sample, Figure 13 shows that the prediction was mainly supported by shape-related descriptors. The strongest positive contributions came from low original_shape2D_

PerimeterSurfaceRatio, low original_shape2D_

Perimeter, an intermediate range of original_shape2D_

Sphericity, and high original_shape2D_

MeshSurface. Smaller contributions were associated with first-order and texture descriptors. This local explanation showed that, for this sample, the model predicted Healthy mainly from geometric regularity and reduced boundary complexity.

**Figure 14:**
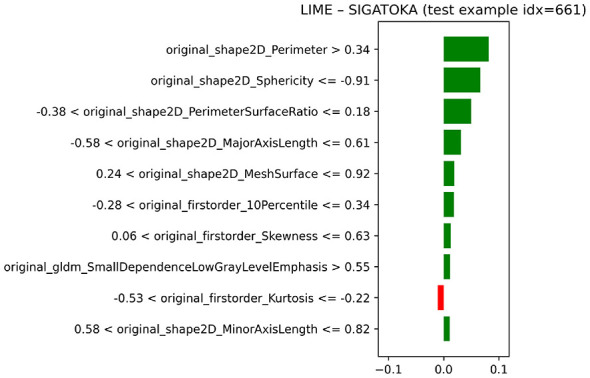
LIME explanation for a representative correctly classified Sigatoka sample.

For a representative Sigatoka sample, Figure 14 shows that the strongest positive contribution came from high original_shape2D_

Perimeter, followed by low original_shape2D_

Sphericity, intermediate original_shape2D_

PerimeterSurfaceRatio, original_shape2D_

MajorAxisLength, and original_shape2D_

MeshSurface. Additional positive contributions came from first-order intensity and GLDM texture descriptors. This local explanation is consistent with the SHAP results, suggesting that Sigatoka predictions were influenced by larger or more irregular segmented regions and associated intensity-texture patterns.

**Figure 15:**
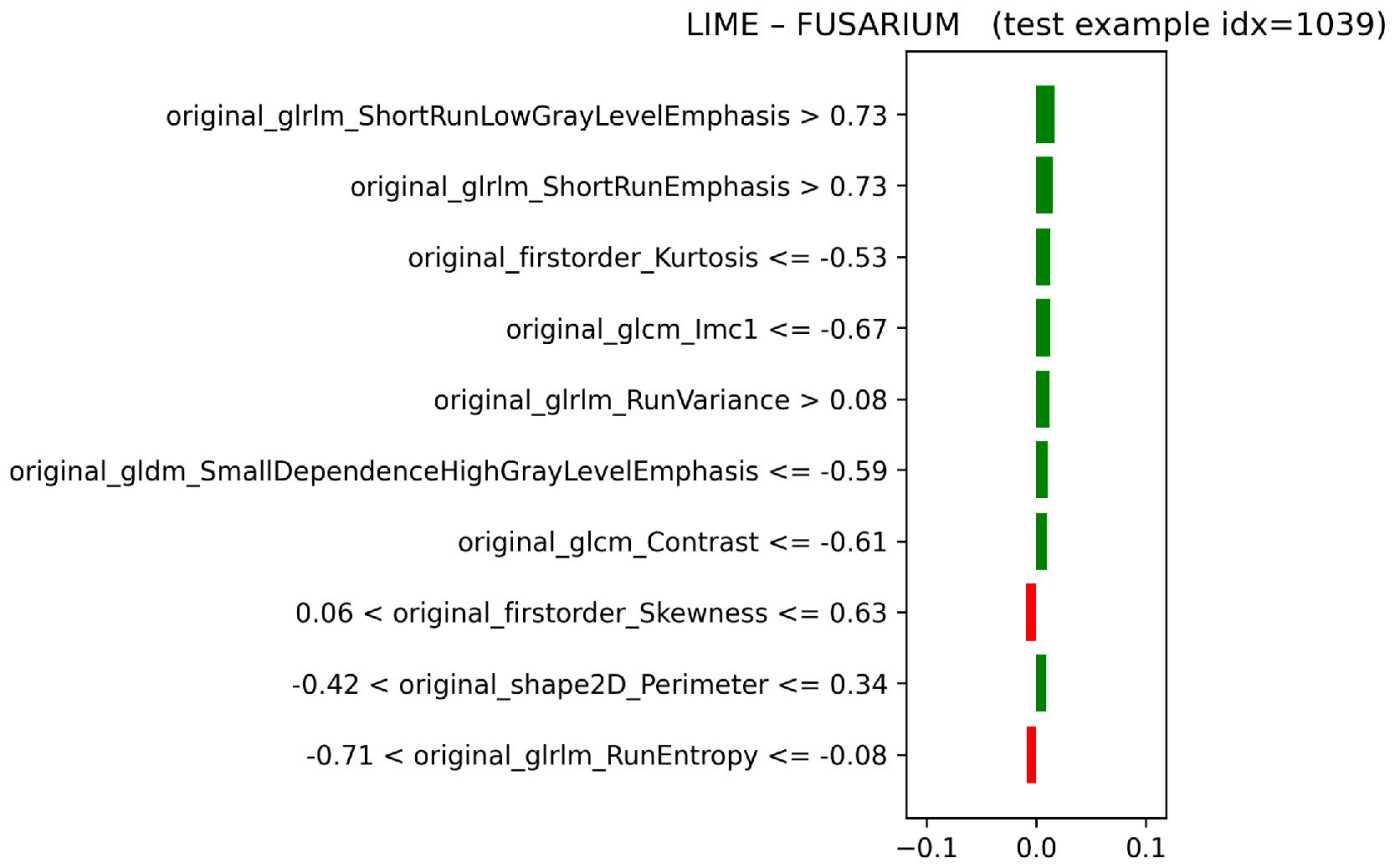
LIME explanation for a representative Fusarium1 sample.

For a representative Fusarium1 sample, Figure 15 shows that local prediction support was dominated by texture descriptors. The strongest positive contributions included high original_glrlm_

ShortRunLowGrayLevelEmphasis and original_glrlm_

ShortRunEmphasis, low original_firstorder_

Kurtosis and original_glcm_

Imc1, high original_glrlm_

RunVariance, and low original_glcm_

Contrast. In contrast, moderate original_firstorder_

Skewness and intermediate original_glrlm_

RunEntropy contributed negatively to this local explanation. The local Fusarium1 prediction was driven primarily by short-run texture structure, gray-level distribution, and local heterogeneity rather than by shape features alone.

The LIME explanations provide local evidence that the radiomics model uses different combinations of descriptors depending on the predicted class. Healthy and Sigatoka examples were mainly influenced by shape-related descriptors, whereas the Fusarium1 example showed stronger dependence on texture and run-length features, showing the interpretability value of the radiomics model, while reinforcing that such explanations describe model behavior rather than causal disease processes.

### 4.7 Accumulated local effects (ALE)

**Figure 16:**
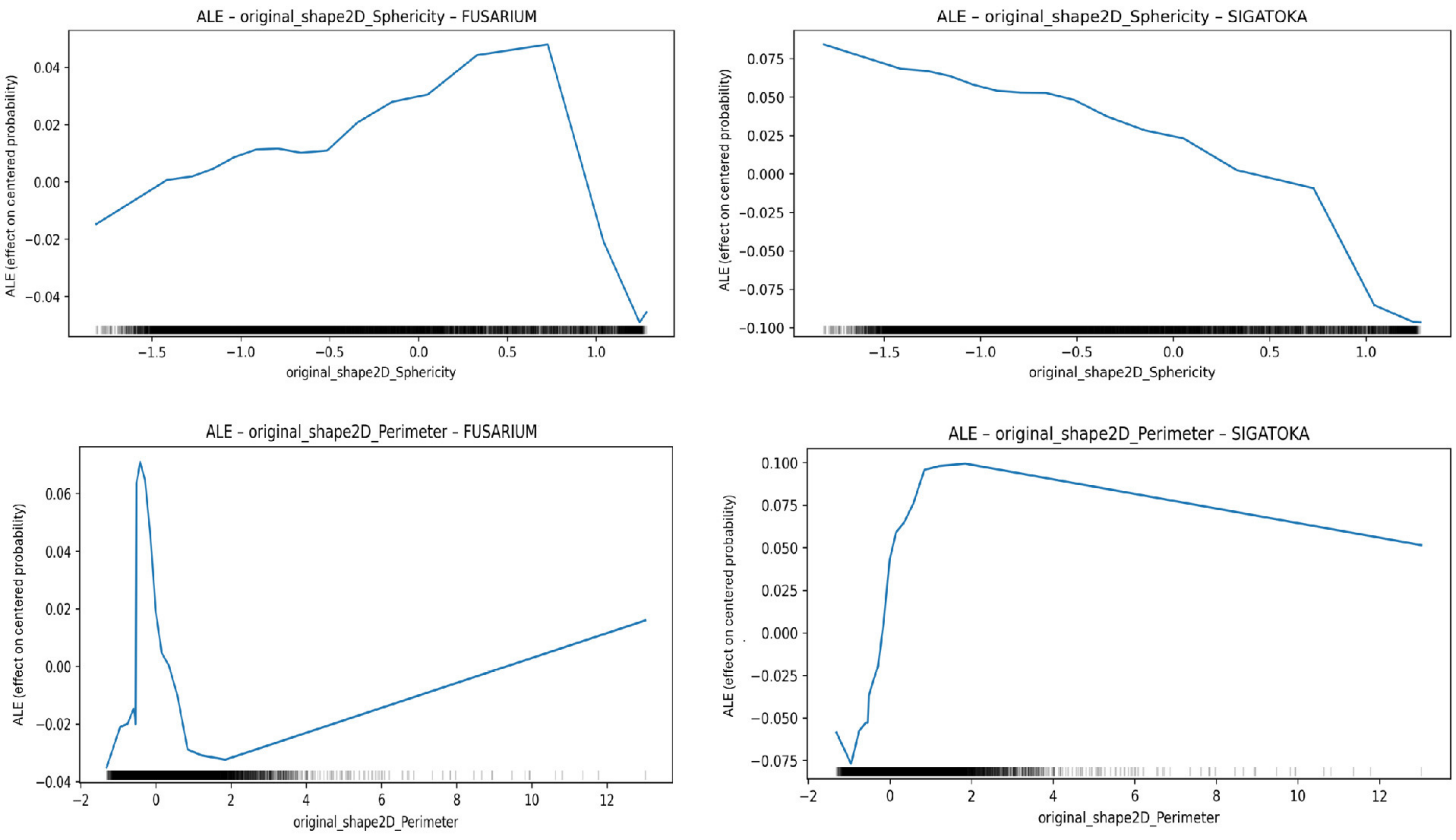
Accumulated local effects analysis illustrating non-linear associations between shape descriptors and disease probability.

Accumulated local effects analysis was also used to inspect non-linear associations between selected shape descriptors and predicted class probabilities. Figure 16 shows that original_shape2D_

Sphericity and original_shape2D_

Perimeter had class-dependent non-linear effects for Fusarium1 and Sigatoka. The direction and magnitude of these effects differed across classes, showing that the shape descriptors influence disease predictions in a non-uniform manner.

These ALE results are exploratory evidence of non-linear feature effects within the trained Random Forest. They do not by themselves prove specific biological interactions among descriptors. Instead, they complement permutation importance, SHAP, and LIME by showing how selected features affect the model output across their observed value ranges.

## 5 Discussion

The experimental results show the feasibility of a calibrated radiomics-based ML framework for banana disease-pattern characterization using RGB imagery. In contrast to end-to-end DL models, the proposed radiomics pipeline relies on explicitly defined quantitative descriptors extracted from leaf-level ROI. This representation allows the model output to be examined through feature-level explanations, calibration analysis, and class-wise error inspection. After HSV-based segmentation and radiomic feature extraction, the dataset comprised *N* = 14, 763 image-level radiomic samples described by 103 initial descriptors, with a moderately imbalanced class distribution. These characteristics motivated the use of leakage-aware partitioning, balanced evaluation metrics, probability calibration, and macro-averaged performance measures.

Among the evaluated radiomics classifiers, the Random Forest achieved the strongest performance. The final calibrated model reached an accuracy = 0.85, balanced accuracy = 0.84, macro-F1 = 0.84, and macro ROC-AUC OvR = 0.95 on the held-out test set. Bootstrap confidence intervals for accuracy and balanced accuracy indicated that the estimates were stable under resampling of the evaluated test set. McNemar testing further showed that the paired selection of error patterns of Random Forest and SVC-RBF differed under the same test partition. Random Forest showed to be the best within the radiomics-based model family.

The inclusion of MobileNetV3, EfficientNet, and ResNet-18 as CNN baselines achieved better performance on the evaluated repositories, with macro-F1 scores above 0.96, whereas the calibrated radiomics Random Forest achieved macro-F1 = 0.84. The radiomics model should not be interpreted as a performance-equivalent substitute for DL in terms of raw predictive accuracy. However, the results support a complementary interpretation. The CNN baselines establish a high-performance image-based benchmark, while the radiomics model provides calibrated probabilities, feature-level interpretability, and traceable quantitative descriptors that can be inspected using permutation importance, SHAP, LIME, and accumulated local effects. This distinction is relevant for phytosanitary decision support, where high classification accuracy must be considered together with calibration, auditability, domain-shift assessment, and external validation before operational deployment.

The high performance of the CNN baselines should also be interpreted cautiously. Although MobileNetV3, EfficientNet, and ResNet-18 demonstrated strong classification capacity, this performance may reflect high separability of the evaluated public repositories, acquisition-specific regularities, background cues, or dataset-dependent visual patterns. Therefore, high internal performance does not eliminate the need for leakage-aware validation and independent testing under different geographic and agronomic conditions. Models trained on images from Tanzania and Bangladesh cannot be assumed to generalize directly to Ecuadorian field conditions without external validation using Ecuadorian images, plant-level metadata, plot-level identifiers, cultivar information, acquisition-device metadata, and confirmatory disease labels.

Probability calibration was relevant because the confidence scores can support threshold-based triage. The reliability diagrams and Brier score decomposition indicated that isotonic calibration improved the reliability of Random Forest probability estimates under the evaluated distribution. These calibrated probabilities may help define different operating thresholds according to the decision objective. A high-sensitivity threshold may be suitable for timely warning or inspection prioritization, whereas a higher-confidence threshold may be required before allocating scarce expert resources. However, calibration does not correct domain shift, does not validate the model externally, and does not replace laboratory confirmation. Its role is to improve the interpretability and reliability of predicted confidence scores within the evaluated data distribution.

The interpretability analyses clarify how the radiomics model generated its predictions. Permutation importance and global SHAP analysis showed that shape-related descriptors, first-order intensity features, and texture descriptors contributed to model performance. Features such as maximum diameter, sphericity, perimeter, perimeter-surface ratio, major axis length, intensity percentiles, and run-length or size-zone descriptors appeared among the most influential variables, showing the model relied on quantitative descriptors related to geometric regularity, intensity distribution, and texture heterogeneity. Class-wise SHAP plots showed that Healthy and Sigatoka predictions were influenced strongly by shape descriptors, whereas Fusarium1 predictions exhibited a more visible contribution from texture and run-length features, which are consistent with the intended role of radiomics as a feature-level interpretable representation.

The LIME explanations provided complementary sample-level evidence. In representative Healthy and Sigatoka cases, local predictions were mainly influenced by shape descriptors, such as perimeter, sphericity, mesh surface, perimeter-surface ratio, and major axis length. In contrast, the representative Fusarium1 explanation showed stronger dependence on GLRLM and GLCM texture descriptors, including short-run emphasis, short-run low gray-level emphasis, run variance, and gray-level association features. These local explanations showed that the Random Forest did not rely on a single universal descriptor across all classes; rather, different combinations of shape, intensity, and texture features contributed to different predictions. SHAP, LIME, and ALE explained the model behavior rather than causal evidence of disease mechanisms. They support auditability and biological plausibility assessment, but causal validation would require controlled agronomic or pathological experiments.

The accumulated local effects analysis also suggested non-linear relationships between selected shape descriptors and predicted class probabilities. The sphericity and perimeter showed class-dependent effects for Sigatoka and Fusarium1. These results can be useful for understanding how the trained Random Forest responds to changes in specific radiomic descriptors across their observed ranges. However, ALE shows exploratory evidence of non-linear feature effects, and not specific feature interactions.

The HSV sensitivity analysis showed that segmentation coverage remained stable under the tested perturbations of the hue threshold, with mean coverage varying within a narrow range, showing internal stability of the segmentation masks for the evaluated repositories. However, fixed HSV thresholding remains a limitation for deployment under uncontrolled field conditions. Illumination changes, shadows, cultivar differences, camera response, background clutter, overlapping leaves, and canopy structure can all affect color-based segmentation. Therefore, adaptive thresholding, color constancy correction, supervised segmentation, or segmentation models trained on field-specific annotations should be considered in future deployment-oriented work.

### 5.1 Limitations and deployment considerations

This study is motivated by the Ecuadorian phytosanitary context, but the images used for model development were obtained from public repositories collected in Tanzania and Bangladesh. Therefore, the results should be interpreted as a methodological proof-of-concept rather than evidence of direct operational validity in Ecuadorian plantations.

Rapid characterization refers to computational characterization of visible disease-related image patterns; the current data do not establish physiological early infection detection in terms of days post-infection. Also, similar visible symptoms may also result from nutritional deficiencies, water stress, senescence, mechanical injury, or other diseases. Therefore, image-based classification should be interpreted cautiously under field conditions, where multiple biotic and abiotic stress factors may coexist.

Although inference with a trained Random Forest is lightweight, segmentation and radiomic feature extraction add preprocessing cost and would require optimization for low-power or mobile deployment. The current pipeline operates at the single-image or leaf-level ROI scale, whereas real plantation monitoring may involve overlapping leaves, complex canopy structures, mixed disease stages, variable illumination, and cluttered backgrounds. Available public repositories do not provide verified plant-level, plot-level, farm-level, or acquisition-device identifiers, limiting the ability to perform stricter biological-unit or domain-aware validation.

Future work will prioritize external validation using Ecuadorian field images collected under both standardized and uncontrolled acquisition conditions. Datasets will include plant-level and plot-level identifiers, cultivar metadata, geographic origin, imaging device information, environmental conditions, and laboratory-confirmed disease status. Also hybrid CNN-radiomics systems will be explored that combine the predictive accuracy of compact CNNs with the interpretability and calibration advantages of radiomics.

## 6 Conclusion

This work presented a calibrated radiomics-based framework for multiclass banana leaf disease-pattern characterization using RGB imagery. The framework integrates HSV-based leaf segmentation, image-level radiomic feature extraction, training-only feature preprocessing, Random Forest classification, isotonic probability calibration, and complementary explainability analyses based on permutation importance, SHAP, LIME, and accumulated local effects. The final calibrated radiomics Random Forest achieved accuracy = 0.85, balanced accuracy = 0.84, macro-F1 = 0.84, and macro ROC-AUC OvR = 0.95 on the held-out test set, while providing calibrated probabilities and feature-level explanations based on quantitative image descriptors.

Three CNN baselines were implemented to clarify the accuracy–interpretability trade-off. MobileNetV3, EfficientNet, and ResNet-18 achieved good predictive performance on the evaluated repositories, with macro-F1 scores above 0.96, showing that CNNs provide a stronger accuracy benchmark under the current data conditions. The radiomics framework provides an auditable and calibrated reference model whose predictions can be inspected through explicit descriptors related to shape, intensity distribution, and texture heterogeneity.

The interpretability analyses showed that the radiomics model relied on different feature patterns across classes. Global permutation importance and SHAP highlighted the relevance of shape descriptors, first-order intensity statistics, and texture features, while class-wise SHAP and LIME explanations indicated that Healthy and Sigatoka predictions were mainly influenced by shape-related descriptors, whereas Fusarium1 predictions showed stronger contributions from texture and run-length features. ALE analysis further suggested class-dependent non-linear effects of selected shape descriptors. These findings support the value of radiomics for model auditability and biological plausibility assessment, while remaining model-based explanations rather than causal evidence of disease mechanisms.

From an operational perspective, the framework should be interpreted as a decision-support and triage approach, not as an autonomous diagnostic system. In particular, Fusarium1-to-Sigatoka errors may have important agronomic implications because Fusarium wilt requires confirmatory inspection, laboratory testing, quarantine, sanitation, and movement-control measures, rather than treatment as a foliar disease. Calibrated probabilities support threshold-based inspection prioritization, but they do not replace expert assessment or official phytosanitary confirmation.

## Data Availability

The original contributions presented in the study are included in the article/supplementary material, further inquiries can be directed to the corresponding author.
